# Comparison between Match and Training Session on Biomarker Responses in Handball Players

**DOI:** 10.3390/sports11040083

**Published:** 2023-04-12

**Authors:** Zoran Nikolovski, Nikola Foretić, Dario Vrdoljak, Dora Marić, Mia Perić

**Affiliations:** 1 Faculty of Kinesiology, University of Split, 21000 Split, Croatia; 2 High Performance Sport Center, Croatian Olympic Committee, 10000 Zagreb, Croatia

**Keywords:** cortisol, testosterone, alpha-amylase, team sport, contacts, playing time

## Abstract

A variety of loads are placed upon an athlete in team sports (e.g., training, match, or competitions). However, the volume of the training load plays an important role in match success. Therefore, the aim of this study was to compare the differences in biomarker dynamics during the match and during training, and to evaluate if such training represents a good stimulus for an athlete to adapt to match stress. Ten male handball players (average age of 24.1 ± 3.17 years, body height of 1.88 ± 0.64 m, and body mass of 94.6 ± 9.6 kg) took part in this study. Their saliva cortisol, testosterone, and alpha-amylase were sampled during the match and training (sessions of 90 min duration, respectively). The results showed that cortisol had higher values after the match (0.65 µg/dL) than after training (0.32 µg/dL) (*p* = 0.05; ES = 0.39). Testosterone concentrations had a steeper increase during a match (65%) than after training (37%). Alpha-amylase levels did not differ significantly between the match and training (*p* = 0.77; ES = −0.06). Overall, the results showed that the environment of a match was more stressful for the athletes; therefore, a match provoked a stronger endocrine response in the studied markers. Therefore, we concluded that a match seemed to be a stronger trigger for all of the measured biomarker responses.

## 1. Introduction

Training is one of the demands that is necessary for the improvement of an athlete. Accordingly, the load during training should be similar to the load during a competition. Therefore, load monitoring in both activities is of the utmost importance, whereas the management of the load during training has been shown in multiple sports to be effective for boosting performance and preventing injuries [1,2].

Handball is characterized as a physically demanding sport consisting of repeated accelerations, sprints, jumps, shots, and rapid changes in movement and contact among players [3]. Because of its nature, handball tends to set a high workload on an athlete during a match. The work-rate intensity and load are divided between external and internal components. The external load has non-cyclic (passes, shots, jumps, falls, and contact) and cyclic (running, walking, jogging, and cruising) activities [4]. According to previous studies, a handball player will perform 825 activity changes in the course of a match. In addition, an athlete will cover an average of 4370 ± 702 m during a match [5]. Apart from that, the internal loads that have been investigated are largely physiological (heart rate (HR) and blood lactate). Players will spend more than half of a match (53%) at 80% of their maximum heart rate (HR_max_), and they will execute actions at an average of 70.9 ± 6.0% of their maximum oxygen consumption (VO_2max_) and attain a blood lactate concentration of 4.8 mmol/L after a match [3,6]. However, there is a lack of evidence that explains the response of the endocrine system during a handball match and training. Measuring hormonal responses during a handball match and during training may provide better insights into internal loads.

Exercise physically stresses the body and activates the hormonal systems, more precisely, the hypothalamic corticotropin-releasing hormone, the anterior pituitary adrenocorticotropic hormone, and adrenal glucocorticoids. Moreover, physiological and psychological factors influence the adrenocortical response in acute or chronic exercise [7].

The biomarkers that have been previously examined are testosterone, cortisol, and alpha-amylase [8,9]. Testosterone (T) is an anabolic hormone that stimulates skeletal muscle growth. It appears that during competition, T is essential for mobilizing performance capacity [10]. Cortisol (C) expresses the activity of the hypothalamic–pituitary–adrenocortical axis. C is involved in the stress response [5], and is often used as a biomarker of physical and psychological stress functioning in athletes [11]. Alpha-amylase (AA) catalyzes the hydrolysis of starch into smaller carbohydrate molecules, such as maltose [12]. Previously, it was shown that AA increases during exercise [13]. More pronounced effects happen at exercise intensities of >70% VO_2max_ [14]. Additionally, AA reflects the activity of the sympathetic nervous system. Levels of AA are highly related to increases in noradrenalin, and consequently, they reflect an individual’s state of arousal [15]. This finding shows how physical activity may lead to increases in AA levels.

Studies carried out thus far have primarily examined an athlete’s hormonal responses and performance during a handball match. According to Foretic, Nikolovski [9], hormonal changes depend on a player’s position, and they are influenced by the number of contacts rather than playing time. The authors stated that contact and non-contact players showed specific biomarker patterns during a match, and they should be trained differently. Furthermore, it has been shown that T levels increase significantly in the first half of a match and AA levels increase constantly throughout a game [8]. The examined literature showed implications on training intensity that could be determined by the investigation of biomarkers [1,2].

However, there is a lack of evidence emphasizing the usefulness of specific training and stimuli for a match in handball. It is hypothesized that a real competition would generate a greater stress response than a simulated condition. Therefore, the aims of this study were to assess the differences in T, C, and AA dynamics during a match and during training and to evaluate if such training is a good stimulus for an athlete to adapt to match stress.

## 2. Materials and Methods

### 2.1. Participants

Ten handball athletes, all males (3 wings, 4 back players, 1 middle-back player, and 2 pivots) (average age of 24.1 ± 3.2 years, average body height of 1.9 ± 0.6 m, and average body mass of 94.6 ± 9.6 kg) took part in this study. The study was conducted during the competitive season. All players were professional players from the same club participating in the first Qatari handball division and Arab Club Champions Cup. They trained regularly for at least 18 h per week. Participants who took part in this study volunteered and were informed about the purpose of the study. Experimental procedures were completed following the Declaration of Helsinki, and they were approved by the corresponding authors’ institutional research ethics board (Ethics Board Approval No. 2181-205-02-05-18-002).

### 2.2. Procedure (Training and Match)

In this study, data from one training and one match were collected. Both activities were carried out at the same time of day (from 11.00 a.m. to 13.00 p.m.), in the same sports hall with air conditioner-controlled environmental conditions (temperature 22 °C, humidity 35%). The match and training session were time-separated for one week.

The number of physical contacts was counted for each player. Counting involved video reviews by the teams’ performance analysts. Playing time was calculated by a video observation analysis, using the Time Calculator software v10.0 (freetimeconverter.com, accessed on 18 August 2020.), for each player. Body mass (measured in 0.1 kg) and body height (measured in cm) were measured using a stadiometer (Seca 213, Hamburg, Germany) and a digital scale (Seca 769, Hamburg, Germany), respectively.

#### 2.2.1. Training

The training in this study was intended to stimulate the development of the players’ specific and situational endurance capacities. It consisted of 5 parts: (1) a warmup, (2) an attacking circuit, (3) a defensive circuit, (4) a specific polygon for shooting on goal, and (5) a specific small-sided game.

After 10 min of warmup, ten male handball players were divided into five pairs. Each pair spent 1 min performing each exercise. They conducted two specific circuits—eight exercises per circuit—in which they performed defensive and attacking handball elements with a ball. After completion of a circuit, the players ran for two min at a low intensity (120–130 HR) with the aim of increasing their total training volume and recovery. The total working time per circuit was approximately eight minutes.

For the next 5 min, the players undertook a specific endurance polygon. The polygon’s movement directed the players to defend or attack, performing several agility, speed, and explosive power actions (defensive lateral movement with a medicine ball, agility ladders, skipping, change in direction speed, and hurdle hops) before shooting on the goal. Shooting was paired with an active goalkeeper.

The last part of the training was a specific handball small-sided game. Players were divided into two teams of six. Half of the players on each team played on only one half of the court, though in cooperation with the rest of the team on the other half of the court. This game was repeated two times, and every playing interval was 8 min long. Between intervals, the players conducted 2 min of low-intensity running.

**Figure 1:**
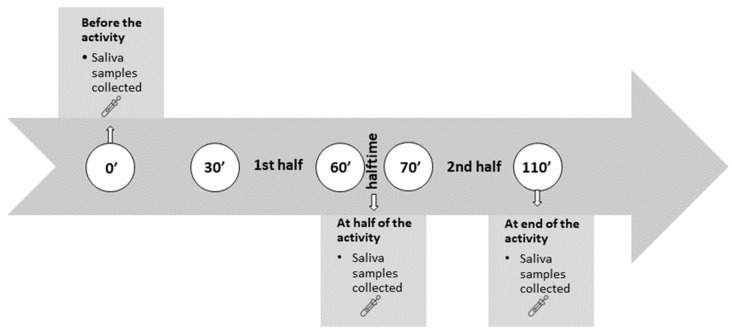
Data collection timeline, 30 min before the match/training, at halftime, directly after the match/training.

The biomarkers were collected before training started (pre-training; 11:00 a.m.), at the halfway point (half training; approximately 12:00 p.m.), and after the training session finished (post-match; 12:50 p.m.) (Figure 1).

#### 2.2.2. Match

Measurements were taken during the handball match. It consisted of two halves of 30 min each, with a standard break of 10 min. In this study, salivary C, T, and AA concentrations were assessed before the match (pre-match; 11:00 a.m.), during the halftime break (halftime; approximately 12:00 p.m.), and after the match (post-match; 12:50 p.m.) (Figure 1).

### 2.3. Sampling and Handling

The sampling of salivary biomarkers was completed accordingly (see Figure 1). The athletes had rested for 48 h after the last training session. After an overnight fast, and without eating a major meal 1 h before sample collection, the athletes were asked to rinse their mouths thoroughly with water 10 min before each sample was collected. SalivaBio Oral Swabs, SOS (Salimetrics LLC, State College, PA, USA), were used. Swabs were placed underneath the tongue on the floor of the mouth for 2 min. Afterwards, the swabs were stored in tubes and placed in the refrigerator immediately. The samples were frozen at below −20 °C until centrifugation, within 2 h after sampling. Before the analysis, the samples were thawed completely and centrifuged at 1500× *g* (3000 rpm) for 15 min. Following centrifugation, assays were performed. Saliva C and T were analyzed with an enzyme-linked immunosorbent assay (ELISA) from Salimetrics LLC (State College, PA, USA), on a microplate reader (Infinite 200PRO, Tecan, Mannendorf, Switzerland). The manufacturer’s instructions and commercially available standards were used to construct standard curves. Additionally, quality control samples were used for all assays (Salimetrics LLC). The average intra-assay coefficient of variation (CV) was 4.6%, with a CV of duplicate analysis of 3.8%, while the assay sensitivity for salivary T was 1 pg/mL. The assay sensitivity for salivary C was 0.007 µg/dL, with an average intra-assay CV of 4.5%. The same batch was used to analyze all samples, to avoid intra-assay variability. Samples for AA were analyzed using a kinetic enzyme assay kit from the same supplier (Salimetrics LLC, State College, PA, USA). The average intra-assay CV was 5.5%. Values were expressed as the AA concentration (U/mL).

### 2.4. Statistical Analysis

Statistical analyses were undertaken on log-transformed data. However, the results in the tables and figures were presented as true-value means and standard deviations. Salivary hormone enzyme activity was corrected for the salivary flow rate. All data were log-transformed to reduce the non-uniformity of error, and normality was tested using the Kolmogorov–Smirnov test procedure. Homoscedasticity was checked by the Levene test. The two-way repeated measurements ANOVA test was used to identify possible differences between the training and match. The differences among the measurements of salivary biomarkers were calculated by the magnitude-based Cohen’s effect size (ES) statistic, with modified qualitative descriptors (trivial ES: <0.2; small ES: 0.21–0.60; moderate ES: 0.61–1.20; large ES: 1.21–1.99; and very large ES: >2.0). The rate change was calculated accordingly and presented as percentage values. Firstly, two measurements between biomarkers were deducted (e.g., C before activity and at the half of the activity). Secondly, the first measurement was divided with the difference obtained by the previous step and then divided with the first measurement (e.g., C before activity). Example as shown:

Difference=C(at the middle of the activity)−C(before the activity)

$$Rate\;of\;change=\frac{Difference}{C\left(before\;the\;activity\right)}$$

The software Statistica ver. 13.0 (Dell Inc., Tulsa, OK, USA) was used for all analyses, and a p-level of 95% (*p* < 0.05) was applied.

## 3. Results

**Figure 2:**
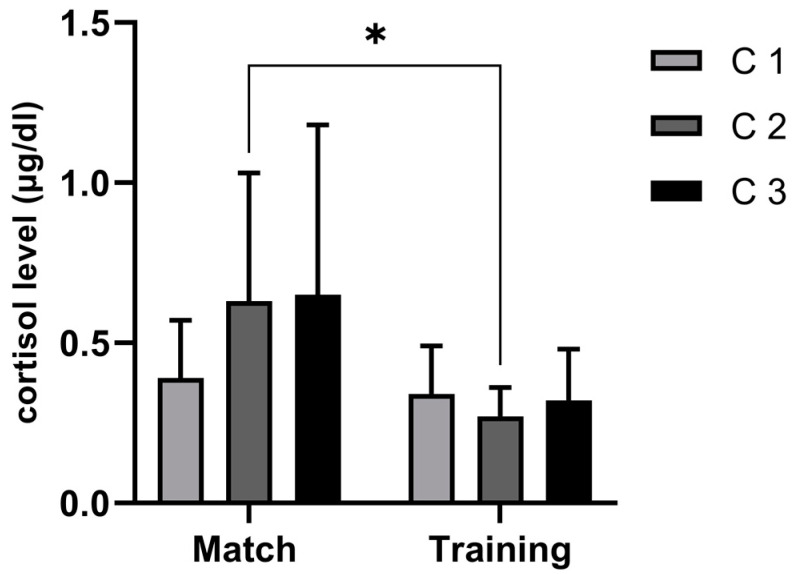
Descriptive statistics for concentrations of cortisol (µg/dL) before the activity (C 1), at the middle (C 2), and at the end (C 3), for both training and match; effect size with significant differences between groups indicated with *.

**Figure 3:**
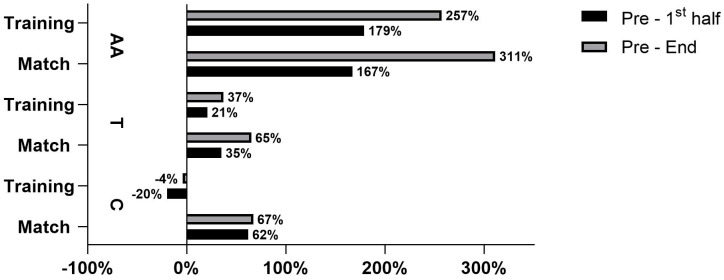
Rate of change for T, C, and AA between match and training’s different time points.

The results showed that the C response was stronger at the third measurement (at the end of the match and after training) than at the beginning and middle of the activities. Further analysis showed that the values were higher in the match environment (0.65 µg/dL) than after training (0.32 µg/dL) (Figure 2). Moreover, Figure 3 presents the rate of change for all the measuring periods. These results showed that the highest percentage rate increase happened between the first and second periods of activity (an increase of 62%).

**Figure 4:**
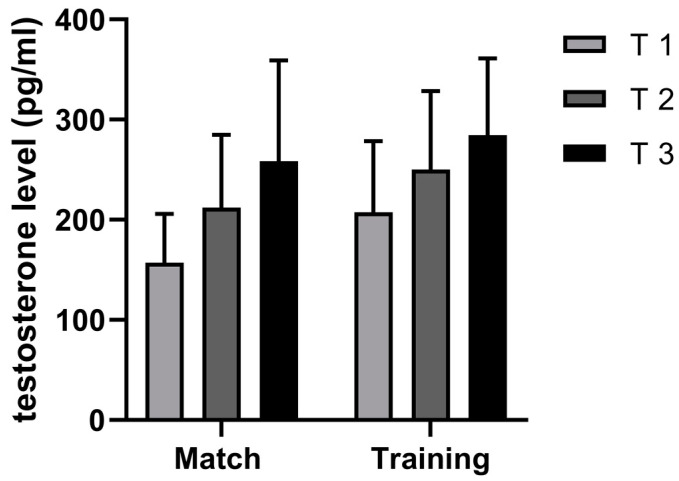
Descriptive statistics for concentrations of testosterone (pg/dL) before the activity (T 1), at the middle (T 2), and at the end (T 3).

T concentrations were shown to be higher before training (207.44 pg/mL) than during a match (157.06 pg/mL) (Figure 4). An analysis of the fold changes showed that T levels rose during both activities, but they were steeper during the match (65%) than during training (37%) (Figure 3).

**Figure 5:**
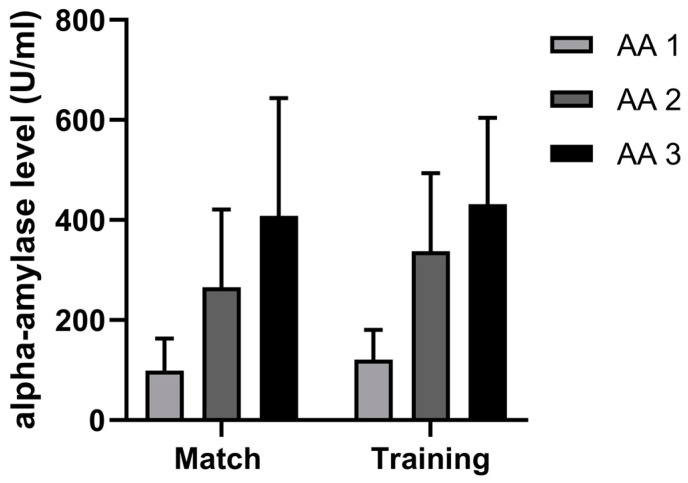
Descriptive statistics for concentrations of alpha-amylase (U/dL) before the activity (AA 1), at the middle (AA 2), and at the end (AA 3).

Contrary to the previously mentioned hormonal response, the AA levels did not differ between the match and training (as seen in Figure 5). In addition, the trend of growth showed that the AA levels increased during both activities, but they increased more during the match (311%), specifically, between the first and second periods (167%) (Figure 3).

**Figure 6:**
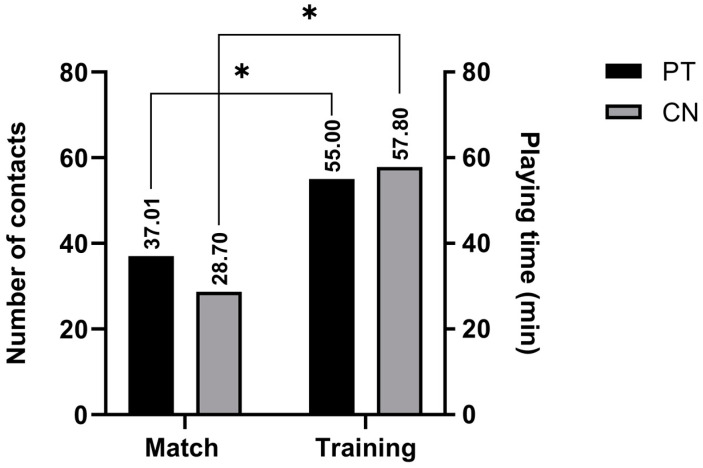
Descriptive statistics for playing time (PT) and number of contacts (CN), for both training and match; effect size with significant differences between groups indicated with *.

Data showed that during the match there were significantly less contacts (CN) (28.10) and playing time (PT) (37.01) than during training (57.81; 55.00, respectively) (Figure 6). Furthermore, a stronger endocrine response was noted in the match (as shown in Table 1).

## 4. Discussion

The main aim of this study was to compare the differences in biomarker dynamics during a handball match and training. Additionally, we evaluated the suitability of particular training as a good stimulus for an athlete to adapt to match stress. Following that, this study had several important findings: (1) the handball match induced stronger endocrine and nervous system reactions compared to specific handball training, (2) physical contact influenced the measured biomarkers more during the match than during training, and (3) C had the strongest effect on the handball match and training stressors.

Generally, the match and training showed similar measured biomarkers’ responses. This implies that the specific handball endurance training employed has to be adopted in order to elicit similar biomarkers’ responses. Specifically, training needs to last longer and produce more heavy and forceful physical contacts.

### 4.1. Difference in C Dynamics between Handball Training and Match

Previous studies on team sport games have shown significant C increases during both training and matches. In some of these studies, there was a stronger C reaction during competition, while in others, the researchers did not report differences in the C dynamics between training and the match [16,17,18].

Gonzalez-Bono, Salvador [18] reported pre- and post-match rises in C levels in male basketball players (3.07 ± 1.31 mmol L^−1^). In another study, Moreira et al. [16] revealed a greater magnitude of C and session RPE responses after an official match compared to a simulated match [16]. Research carried out on soccer players has reported the same dynamics. Additionally, Haneishi et al. [17] found that in female collegiate soccer players, pre-game C concentrations (18.0 ± 10.3 mmol L^−1^) were significantly higher than pre-practice levels (8.3 ± 3.5 mmol L^−1^). Moreover, post-game C responses (53.1 ± 33.9 mmol L^−1^) have been shown to be higher than post-practice C responses (22.4 ± 13.8 mmol L^−1^) [16]. Researchers have published inconsistent findings for volleyball and rugby [11,19,20]. In the study by Elloumi et al. [20], the authors reported sharp increases in C levels during a rugby match (approximately 2.5-fold compared to resting values), although C levels returned to basal values within 4 h after the match concluded [20].

Regarding acute C responses in handball, studies are lacking. However, there is some evidence of changes in C levels in the course of the match and training [8,21,22,23]. In particular, Chatzinikolaou, Christoforidis [24] presented C levels that only increased post-game (by approximately 25%). Mariscal, Vera [23] reported that playing time had a large influence on salivary C increases in female handball players.

In our study, C levels increased throughout a match (67%), whereas during training, the response was negative (−4%). Further, for the second measurements, between training and the match, medium effect sizes were observed (ES = 0.53). These results suggested that the players perceived much greater amounts of stress during competition, even though the physical load during training was higher (i.e., significantly more contacts and longer playing times). The physical contacts in handball are fairly strong and are combined with other explosive actions such as sprinting, jumping, shooting, and changes in direction [23,25]. Therefore, the assumption was that players were exposed to a much harder physical load during a match. Furthermore, higher levels of C after a match may suggest that apart from playing time and number of contacts, the match importance presented a higher psychological challenge for the players compared to training [18,24,26]. Thus, Souza, Beltran [24] stated that both the emotional and psychophysiological indices of stress for athletes from various sports are higher before competition than they are before training. Moreover, for a variety of sports (including handball), the rate of physical load has been shown to be related to increases in C levels [27]. Therefore, it can be concluded that numerous factors may influence the C response in a stressful environment, such as a handball match.

### 4.2. Differences in T Dynamics between Handball Training and Match

Success in some men’s team sport competitions has been associated with significant increases in T levels [20,28]. As demonstrated previously, high-intensity physical activity influences the T response [29]. However, in high-contact sports, athletes express different responses, according to different authors. In a study by Barnes, Mundel [29], rugby players’ responses increased by 33%, while others experienced a decrease of 44% [30]. These findings imply that other factors influence the T response. Furthermore, during rugby training protocols, athletes experienced an increase in T levels for two different types of training, to be exact, and a 23% increase was visible after high-intensity training, while a 55% increase occurred in a wrestling and situational environment. Obviously, T levels may increase due to high external loads and physical contact [31]. Moreover, according to Aguilar, Jiménez [32], field hockey players showed a negative correlation between lactate levels and T changes. In particular, the players that perceived higher levels of physical exertion showed higher inhibitory influences in their post-game T levels. According to some authors, the winners in a competition show T increases while the losers exhibit T decreases [18,33], which show the high impact of psychological factors on the T response. Playing position is also shown to be a possible influence on the T response. Various studies have shown how players in positions that are more exposed to physical contact (e.g., centers in basketball and defense in field hockey) release higher T levels [32,34].

Obviously, the T response can be affected by different match elements, such as playing positions, players’ roles, physical demands, and/or psychological strains. All of these elements draw our attention to expect a stronger T reaction during competition than during training. At first, the present study did not show this kind of T dynamic. Statistically significant differences were not found in T concentrations between training and the match (Figure 3). Interestingly, T concentrations before training were 32.07% higher than before the match, and this difference remained until the end of the activity. T levels were higher at the middle point of an activity (17.97%) and at the end of an activity (10.45%) when comparing both the training session and match. The lessening of the differences throughout the activities raised interest to analyze the rates of change between the beginning and the end of the activities (both training and match). When analyzing the rates of change, it was noticed that changes in T concentrations were more influenced by the match than by training demands (Figure 5). Between the beginning and the end of a match, T levels rose by 65%. This is almost double the 37% rate of change measured between the beginning and the end of training. Although the players played 18 min longer and conducted 29.1 more contacts during training sessions, their T responses were stronger during the match. From these data, it can be speculated that the physical contact intensity and playing time volume during training were not sensitive enough components for the expected T response rate of change. Since other parameters of the internal/external loads of the players (RPE, HR, La, or LPS data) were not measured, the hypothesis cannot be confirmed that the match induced significantly stronger T responses than training. Nonetheless, the dynamics of the handball match and training appeared to be different [35,36]. Studies conducted on handball players have shown that a handball match is full of high-intensity activities (jumps, shots, sprints, direction changes, etc.) and stressful situations, such as severe physical contact [25]. Playing intensity and the high-contact nature of a handball match play important roles in injury rates. Injury rates are significantly higher during a match (i.e., 8.3–14.3 injuries/1000 h) than during training (i.e., 0.6–4.6 injuries/1000 h) [37]. Therefore, injury-threatening situations during handball matches can trigger stronger T responses than during training. According to Gleeson et al. [38], T acts on specific substrates in the brain to increase aggression and motivation during competition [38]. In this study, even though the number of contacts and the playing time were higher and longer during training, the rates of change in the T responses throughout the match were higher, indicating that the injury-threatening setting of a match can strongly influence aggressive and protective behaviors, resulting in a stronger T response.

One of the reasons we can speculate for the differences between T values before training and matches might be due to different times of waking-up of the players. T levels drop throughout the day after waking-up, and these values are faster dropping if pre-waking values of T were higher [39]. Therefore, our data show that the T trend of change was similar even though the baseline values were different. The lack of significant differences in T concentrations between training and the match might be due to the similarity of the stress produced by training and the match.

### 4.3. Difference in AA Dynamics between Handball Training and Match

AA has been shown to be an important marker of stress and a factor that can be used to differentiate the levels of stress between the match and training. Previous studies have shown that AA has a significantly higher response in a match compared to training [13,40,41]. Kivlighan and Granger [13] demonstrated a 156% increase in AA levels 20 min after an ergometer competition. In team sports, when studies were carried out only during training sessions, the evidence suggested that there were significant increases in AA [19]. Additionally, researchers have reported that AA increases during a match [42].

In the present study, it was demonstrated that there were high increases in AA responses for both the match (311%) and training (257%). Furthermore, AA levels rose constantly throughout the match and training, and the highest increases were detected between the first and second measurements. These results do not corroborate previous findings, where it has been demonstrated that the match environment boosts AA more than training [40]. Data should be put in a specific context and explored differently. Specifically, the playing time and contact number during the training and match were 50% and 100% higher, respectively. As demonstrated previously, the playing time and number of contacts influenced the AA response during a match [8]. As such, it can be speculated that a longer playing time and a higher number of contacts are needed during training to elicit an AA response similar to that seen during a match. This has an important implication for training organizations, as it appears that the load during training has to be much higher in order to mimic that expected at a match. The existence of some confounding factors cannot be neglected, such as psychological stress, which was not analyzed in the present study.

### 4.4. Limitations

This study has several limitations. Above all, internal and external load parameters that would help to better understand the endocrine and nervous system responses during the handball match and training were not measured. Furthermore, the small sample of athletes and activities (only one training session and one match) did not provide the opportunity to use a more relevant statistical analysis and draw more significant conclusions about the measured biomarkers and their relationships with handball training and match demands. Another study limitation is the research was of only male athletes, since a literature review suggested significant differences in the biomarker response during competition, specifically in female athletes’ AA [43].

## 5. Conclusions

This study showed similar biomarker dynamics during handball training and matches. This led us to conclude that a match appeared to be a stronger trigger of all the measured biomarker responses. This is most likely due to the higher intensity of play and the more significant psychological demands that occur during a handball match. The biggest differences were noticed between training and the match in C response levels, while both T and AA showed similar dynamics and differences between training and the match. Nevertheless, according to these study results, handball conditioning experts should consider that the load during training has to be much higher in order to mimic that expected at a match.

## Figures and Tables

**Table 1:** Descriptive statistics for measured salivary biomarkers with differences obtained by analysis of variance (ANOVA) and effect size (ES).

Variables	Mean	Min	Max	SD	ANOVA	ES	Skew.	Kurt.	Max D	K-S
C 1 m	0.39	0.14	0.66	0.18	0.46	0.15	0.06	−1.19	0.15	*p* > 20
C 1 t	0.34	0.23	0.75	0.15			2.52	7.01	0.29	*p* > 20
C 2 m	0.63	0.29	1.51	0.40	0.14	0.53 *	1.66	1.88	0.31	*p* > 20
C 2 t	0.27	0.16	0.42	0.09			0.71	−0.17	0.19	*p* > 20
C 3 m	0.65	0.23	1.78	0.53	0.05 ^¥^	0.39	1.52	1.26	0.29	*p* > 20
C 3 t	0.32	0.17	0.63	0.16			0.94	−0.11	0.16	*p* > 20
T 1 m	157.06	98.84	235.89	48.93	0.00 ^¥^	−0.38	0.32	−1.53	0.20	*p* > 20
T 1 t	207.44	141.33	354.53	71.02			1.33	0.92	0.29	*p* > 20
T 2 m	212.02	139.09	340.19	72.75	0.17	−0.24	0.83	−0.65	0.21	*p* > 20
T 2 t	250.12	145.10	402.72	78.43			0.83	0.52	0.24	*p* > 20
T 3 m	258.43	106.90	420.81	100.70	0.43	−0.14	0.03	−1.15	0.19	*p* > 20
T 3 t	284.57	181.92	430.36	76.62			0.37	0.03	0.12	*p* > 20
AA 1 m	99.29	37.26	225.17	63.55	0.37	−0.17	1.11	0.02	0.26	*p* > 20
AA 1 t	121.09	43.92	215.33	59.08			0.18	−1.27	0.14	*p* > 20
AA 2 m	265.35	96.60	609.22	155.83	0.14	−0.23	1.38	2.38	0.21	*p* > 20
AA 2 t	337.55	144.09	667.18	156.06			0.86	0.95	0.16	*p* > 20
AA 3 m	408.53	114.50	853.79	235.38	0.77	−0.06	0.61	−0.24	0.18	*p* > 20
AA 3 t	431.94	134.55	688.64	172.06			−0.25	−0.74	0.17	*p* > 20
PT m	37.01	14.17	53.08	11.86	0.00 ^¥^	−0.73 *	−0.51	0.29	0.17	*p* > 20
PT t	55.00	55.00	55.00	0.00			-	-	1.00	*p* < 01
Total CN m	28.70	4.00	65.00	20.69	0.00 ^¥^	−0.69 *	0.43	−1.05	0.17	*p* > 20
Total CN t	57.80	50.00	64.00	5.33			−0.26	−1.86	0.22	*p* > 20

C, cortisol; T, testosterone; AA, alpha-amylase; PT, playing time; CN, contacts; m, match; t, training; 1, before the activity; 2, in the middle of the activity; 3, after the activity; SD, standard deviation; K-S, Kolmogorov–Smirnov test; ES, effect size with significant differences between groups indicated with *; ^¥^, statistical significance of *p* < 0.05.

## Data Availability

Not applicable.
